# The impact of cesarean delivery on infant DNA methylation

**DOI:** 10.1186/s12884-021-03748-y

**Published:** 2021-03-30

**Authors:** Qian Chen, Yanhong Ming, Yuexin Gan, Lisu Huang, Yanjun Zhao, Xia Wang, Yongjie Liu, Jun Zhang

**Affiliations:** 1 Ministry of Education-Shanghai Key Laboratory of Children’s Environmental Health, Xinhua Hospital, Shanghai Jiao Tong University School of Medicine, 1665 Kongjiang Road, Shanghai, 200092 China; 2 Department of Obstetrics and Gynecology, Hospital of Obstetrics and Gynecology, Fudan University, Shanghai, 200011 China; 3 Department of Pediatrics, Xinhua Hospital, Shanghai Jiao Tong University School of Medicine, Shanghai, 200092 China; 4 Department of Child Health Care, Shanghai Children’s Hospital, Shanghai Jiao Tong University, Shanghai, 200040 China

**Keywords:** Cesarean deliveries on maternal request, Cord blood, DNA methylation, Perinatal epidemiology

## Abstract

**Background:**

Mounting evidence suggests that cesarean delivery may have a long-lasting effect on infant health. But the underlying mechanisms remain unclear. This study aims to examine whether cesarean delivery on maternal request without any medical indications (CDMR) impacts DNA methylation status in the umbilical cord blood of the infant.

**Methods:**

A cross-sectional study was conducted in Shanghai, China. A total of 70 CDMR and 70 vaginal deliveries (VD) were recruited in 2012. The cord blood DNA methylation status was measured in 30 CDMR and 30 VD newborns using Illumina Infinium Human Methylation 450 K BeadChip. To validate the results, the cord blood DNA methylation status was measured in another 40 CDMR and 40 VD newborns using targeted bisulfite sequencing assay. A total of 497 CpG sites from 40 genes were included in the analysis.

**Results:**

A total of 165 differentially methylated positions (DMPs) exhibited differences in DNA methylation by 10% or more between the CDMR and VD groups, many of which were related to the development of the immune system. Based on the targeted bisulfite sequencing assay, 16 genes (16/22, 72.7%) had higher methylation level in the CDMR group than the VD group. Among them, 5 genes were related to the immune system. After considering the estimation of cell type proportions, there was few significant differences in DNA methylation between CDMR and VD groups.

**Conclusions:**

The DMPs identified between CDMR and VD groups might be largely explained by the cell type proportions. Further studies are needed to examine DNA methylation in each cell type separately.

**Supplementary Information:**

The online version contains supplementary material available at 10.1186/s12884-021-03748-y.

## Background

Birth is probably the most dramatic experience in the whole life. After labor for several hours, a totally dependent fetus must rely on his body systems to survive in the outside world. Labor, therefore, may not only be a physical process of birth but also a physiological process to prepare the fetus to survive independently [1]. Pre-labor cesarean delivery (CD) interrupts this natural process and, therefore, might have important health impacts. However, our understanding in this aspect is very limited.

China is one of the countries with the highest CD rates in the world [2]. Moreover, almost half of CDs were due to maternal preference or other social factors instead of clinical reasons [3]. The overuse of CD has raised public health concerns [4]. More and more evidence indicate that CD may have long-term impacts on not only maternal but also child health. For example, epidemiologic studies showed that CD may increase risks of childhood asthma and other allergic disorders [5–7], and obesity [8]. But the biological mechanisms remain unclear.

Among possible biological links between CD and childhood disorders, physiological effects of stress caused by strong uterine contraction during labor may play an important role. Normal epigenetic status may also be altered in CDs compared with vaginal deliveries (VD) [9–11]. The epigenetic alterations not only change the expression of the functional genome and mediate adaptations to a dynamic environment but also retain its stability for the cells lifetime, even after divisions [12, 13]. Therefore, DNA methylation may be one of the possible mechanisms that explain why the mode of delivery may have long-term effects on the offspring’s health.

Our study aimed to investigate the association between CD and DNA methylation status in the cord blood. To avoid any potential confounding effects of maternal and/or fetal complications on the association, we selected women who had CD due to maternal request without any medical indication (CDMR) as the exposed group and normal VD as the non-exposed group.

## Methods

### Study population

In 2012, we recruited 70 women who had CDMR and 70 women who had VD from Xinhua Hospital affiliated to the Shanghai Jiao Tong University School of Medicine in Shanghai, China. Women who had CDMR and VD were matched on their age at delivery (within 5 years), pre-pregnancy body mass index (BMI) (within 1 kg/m^2^), infant birthweight (within 300 g), and gestational weeks at delivery (within 1 weeks). A research nurse introduced the project to the women and a written informed consent was obtained prior to delivery. The ethics approval was obtained from the Ethics Committee of Xinhua Hospital affiliated to the Shanghai Jiao Tong University School of Medicine. The women were asked of a wide array of questions such as age, education, occupation, family income, alcohol use, smoking and exposure to second hand smoke during pregnancy. At birth, umbilical cord blood samples were taken and newborn anthropometric measures were recorded. Detailed obstetric information was extracted from medical records after delivery.

### Sample collection and preparation

Blood samples were collected at delivery by double clamping the umbilical cord at the placental end and the infant end. The whole blood was centrifuged at 4 °C (10 min, 1500 g), then buffy coat was collected in BD EDTA tubes (QIAamp DNA Mini Kit; Qiagen, Hilden, Germany) and stored at − 80 °C until DNA extraction.

### Genome-wide, locus-specific DNA methylation analysis in cord blood

The 30 CDMR and individually matched 30 VD samples were randomly selected for methylation analysis. Genomic DNA was isolated from 200 μL whole blood for each sample. Then the bisulfite treatment of genomic DNA was performed with an EZ DNA Methylation kit (Zymo, Irvine, CA) in accordance with the protocol. Quantitative DNA methylation were measured using Illumina Infinium Human Methylation 450 K BeadChip (Illumina, San Diego, CA). More than 485,000 cytosine phosphate-guanine (CpG) sites were included in each chip. The arrays were scanned using an Illumina iScan with initial quality review using GenomeStudio software. The level of methylation was expressed as a “beta” value (β-value), ranging from 0 (no cytosine methylation) to 1 (complete cytosine methylation). β-values are reported as percentages. Quality control measures were routinely taken and the details were described elsewhere [14]. CpG sites located at known single-nucleotide polymorphisms and on the X and Y chromosomes (list provided by the manufacturer) were discarded to avoid potential confounding by single-nucleotide polymorphisms and gender.

Differential DNA methylation positions (DMPs) were identified using the GenomeStudio methylation module v1.8 [15]. DMPs were defined as those that exhibited a 10% or greater difference in the DNA methylation between the CDMR and VD groups at p < 0.05 [11]. By using the annotation provided by Illumina, we summarized the DMP information over genes by computing the number of probes for each gene, the number of DMPs, and how many DMPs were hypermethylated or hypomethylated. In addition, gene ontology (GO) terms and Kyoto Encyclopedia of Genes and Genomes (KEGG) pathways were used to represent methylated genes. To investigate the functional relevance of the selected DMPs, we used the online DAVID bioinformatics resource to test for enrichment in gene ontology biological processes, and we used the Benjamini-Hochberg procedure to control for false discoveries [16].

Moreover, we estimated the proportions of different cell types using FlowSorted.CordBloodCombined.450 k package in R. This package was derived from 263 umbilical blood cell references. Details of this package and the accompanied data are shown elsewhere [17].

### Targeted bisulfite sequencing assay

To validate the results of the methylation chip, we used the rest 40 CDMR and individually matched 40 VD samples. Targeted bisulfite sequencing assay DNA extraction and bisulfite conversion were performed as previously described. Considering the genes function and the design of upstream and downstream primers, we selected 40 genes to validate the results of 450 K BeadChips. Then the primers were carefully designed in order to detect them in a panel (Additional file Table S1). The net-PCR was performed firstly to amplify the targeted DNA sequence. Then, the target genes were sequenced by Illumina Hiseq 2000. All samples achieved a mean coverage of 600 base-pairs. More CpG sites around the target CpG sites were found in the validation experiments. Finally, 497 CpG sites were included in the validation assay. The methylation level of each CpG site was calculated as the percentage of the methylated cytosines over total tested cytosines. The average methylation level was calculated using methylation levels of all measured CpG sites within the gene.

### Identification of CpG sites associated with diseases

The comparative toxicogenomics database (CTD, http://ctdbase.org/) was used to find integrated chemical-gene, chemical-disease, and gene-disease interactions to generate expanded networks and predict novel associations [18]. We used the database to analyze relationships between CpG sites and diseases. Then, the relationships between CpG sites and diseases were identified.

### Statistical analysis

Socio-demographic characteristics were compared between CDMR and VD using median values with ranges. When analyzing the effects of covariates on DNA methylation, beta-values were converted to M-values. The methylation status was compared between CDMR and VD using t-test and multiple linear regression model. In addition, we applied the empirical Bayes smoothing to the standard errors using the R Bioconductor limma pipeline to detect DMPs on M-value. For multiple testing, FDR correction was performed. A significance level of 0.05 is used in the analyses. SAS version 9.4 for Windows (SAS Institute, Inc., Cary, NC) was used to compare subjects’ characteristics while R was used to analyze the locus specific methylation.

## Results

Table 1 shows that there were no significant differences in maternal characteristics, gender distribution or birthweight between the VD and CDMR groups except that the VD group had slightly more multipara than the CDMR group.

**Table 1:** The characteristics of the study group in the DNA methylation analysis by delivery mode

	DNA methylation chip analysis			DNA methylation validation analysis		
	VD (*n* = 30)	CDMR (*n* = 30)	p	VD (*n* = 40)	CDMR (*n* = 40)	p
Gestational age (weeks)			1.00			0.14
37	2 (6.7)	1 (3.3)		2 (5.0)	1 (2.5)	
38	8 (26.7)	7 (23.3)		7 (17.5)	11 (27.5)	
39	14 (46.7)	15 (50.0)		12 (30.0)	18 (45.0)	
≥ 40	6 (20.0)	7 (23.3)		19 (47.5)	10 (25.0)	
Birthweight (grams)	3281 ± 287	3392 ± 262	0.12	3392 ± 295	3430 ± 295	0.57
Parity			0.49			0.24
0	28 (93.3)	30 (100)		37 (92.5)	40 (100)	
≥ 1	2 (6.7)	0 (0)		3 (7.5)	0 (0)	
Maternal age (years)			0.62			0.25
< 25	4 (13.3)	3 (10.0)		3 (7.5)	2 (5.0)	
25–29	14 (46.7)	18 (60.0)		21 (52.5)	21 (52.5)	
30–34	7 (23.3)	7 (23.3)		16 (40.0)	13 (32.5)	
≥ 35	5 (16.7)	2 (6.7)		0 (0)	4 (10.0)	
Pre-pregnancy BMI (kg/m^2^)			0.73			0.74
< 18.5	6 (20.0)	4 (13.3)		8 (20.0)	5 (12.5)	
18.5–24.9	24 (80.0)	25 (83.3)		29 (72.5)	30 (75.0)	
25.0–29.9	0 (0)	1 (3.3)		3 (7.5)	4 (10.0)	
≥ 30	0 (0)	0 (0)		0 (0)	1 (2.5)	
Han ethnicity	29 (96.7)	28 (93.3)	1.00	40 (100)	39 (97.5)	1.00
Male fetus	10 (33.3)	16 (53.3)	0.19	22 (55.0)	23 (57.5)	1.00
Maternal education			0.24			0.09
High school and below	2 (6.7)	2 (6.7)		8 (20.0)	2 (5.0)	
College	21 (70.0)	26 (86.7)		31 (77.5)	37 (92.5)	
Postgraduate	7 (23.3)	2 (6.7)		1 (2.5)	1 (2.5)	

Note: Data presented are mean ± standard deviation, or n (%)

Fisher’s Exact test, or Student’s t test was used to compare the difference of characteristics between VD and CDMR

A total of 180 DMPs exhibited differences in DNA methylation by 10% or more between the two groups. After 15 DMPs located on the sex chromosomes were excluded, 165 DMPs still remained (*P* < 0.05) (Additional file Table S2). Among them, 121 (73.3%) were associated with known genes, and the majority of the DMPs were in gene bodies. Among them, 61.2% (101 of 165) were found to be hypermethylated in the CDMR as compared to VD (Fig. S1 in Supplemental Material). After the conversion of Beta-values to M-values, we used multiple linear regression to validate the present results. The *p* values and FDR values were shown in Table S4 in the Supplemental Material.

The estimate proportions of cell type in our samples were shown in Table S5 in the Supplemental Material. These cell types contained B cells, CD4 T cells, CD8 T cells, granulocytes, monocytes, natural killer cells (NK), and nucleated red blood cells (nRBC). According to the estimate, the majority of cord blood was granulocyte, followed by CD4 T cells. Multiple linear regression model adjusting for the estimate proportions of cell type was used to avoid potential confounding effect of the cell type (Additional file Table S4).

A total of 20 GO terms were enriched based on the 450 K analysis (Table 2). Many of them have been reported to be associated with the immune system. Furthermore, the DMPs were overlapped with 10 KEGG pathways that are potentially associated with the immune system (Table 3).

**Table 2:** Significantly enriched gene ontology (GO) terms of differentially methylated genes in VD vs CDMR group based on the 450 K analysis

GO Term ID	GO Term	Count ^a^	*P* value	Group
GO:0032395	Major histocompatibility complex (MHC) class II receptor activity	5	2.99E-06	MF ^b^
GO:0002504	Antigen processing and presentation of peptide or polysaccharide antigen via MHC class II	5	3.33E-05	BP ^c^
GO:0042613	MHC class II protein complex	5	3.47E-05	CC ^d^
GO:0042611	MHC protein complex	5	0.0005	CC
GO:0019882	Antigen processing and presentation	5	0.001	BP
GO:0006955	Immune response	11	0.01	BP
GO:0045202	Synapse	8	0.01	CC
GO:0044456	Synapse part	6	0.02	CC
GO:0003779	Actin binding	6	0.03	MF
GO:0030054	Cell junction	8	0.05	CC

^a^ Count, number of DMPs clustered in the GO term; ^b^ MF, molecular function; ^c^ BP, biological process; ^d^ CC, cellular component

**Table 3:** Significantly enriched Kyoto Encyclopedia of Genes and Genomes (KEGG) terms of differentially methylated genes in VD vs CDMR group based on the 450 K analysis

KEGG Term ID	KEGG Term	Count ^a^	P value
hsa05310	Asthma	6	3.11E-07
hsa05330	Allograft rejection	5	3.31E-05
hsa05332	Graft-versus-host disease	5	4.57E-05
hsa04940	Type I diabetes mellitus	5	6.15E-05
hsa04672	Intestinal immune network for IgA production	5	1.14E-04
hsa05320	Autoimmune thyroid disease	5	1.33E-04
hsa05322	Systemic lupus erythematosus	6	1.45E-04
hsa05416	Viral myocarditis	5	4.81E-04
hsa04514	Cell adhesion molecules (CAMs)	6	5.57E-04
hsa04612	Antigen processing and presentation	5	8.70E-04

^a^ Count, number of DMPs clustered in the KEGG term

Additionally, we applied the empirical Bayes smoothing t using the R Bioconductor limma pipeline to detect DMPs on M-value. With the threshold of FDR (< 0.01) and a difference in the mean β value |Δβ| ≥ 5% [19], 1208 DMPs were identified after adjusting the covariates. Under the threshold difference in the mean β value |Δβ| > 10%, 4 DMPs were identified and shown in Table S6.

In the targeted bisulfite sequencing assay, the mean coverage of sequencing was 600 base-pair, 497 CpGs around the selected 40 DMPs were identified finally. There were 212 CpG sites around 25 DMPs were different between the CDMR and VD groups. The methylation level of 148 CpG sites (148/212, 69.8%) was higher in the CDMR group than in the VD group. The methylation level of 64 CpG sites (64/212, 30.2%) was lower in the CDMR group than in the VD group. The average methylation level of all CpG sites was calculated as the methylation level of the genes (Additional file Table S3). A total of 22 (55%) genes showed differences between CDMR and VD. Six genes (6/22, 27.3%) had lower methylation status in CDMR group than in VD group, and 16 genes (16/22, 72.7%) had higher methylation level in CDMR group than VD group (Table 4). Among them, five genes were related to the immune system, including phosphatidylinositol- 4,5- bisphosphate 3- kinase catalytic subunit delta (PIK3CD), CXXC finger protein 5 (CXXC5), lymphotoxin β receptor (LTBR), Spi-1 proto-oncogene (SPI1) and serine protease inhibitor B9 (SERPINB9).

**Table 4:** Differentially methylated genes in VD vs CDMR group

Order	Target	*P*-value	MethylDiff
1	DGKA_cg07679948_	< 0.0001	−0.04
2	ZFP36L1_cg10099732_	< 0.001	−0.04
3	RCAN3_cg25140783_	< 0.01	−0.04
4	RASA3_cg21261158_	< 0.05	−0.03
5	SERPINB9_cg01345354_	< 0.001	−0.02
6	PIK3CD_cg23098018_	< 0.001	−0.01
7	TUBGCP6_cg19851816_	< 0.001	0.02
8	SPI1_cg15982099_	< 0.001	0.02
9	MYL9_cg18660345_	< 0.05	0.02
10	CXXC5_cg19628988_	< 0.001	0.03
11	TSPOAP1_cg17525495_	< 0.001	0.03
12	LTBR_cg19476647_	< 0.001	0.03
13	SLC12A7_cg18997983_	< 0.01	0.03
14	DENND3_cg14480530_	< 0.05	0.03
15	DOC2B_cg14984781_	< 0.05	0.04
16	PRTN3_cg19787694_	< 0.0001	0.05
17	AGTRAP_cg25467652_	< 0.001	0.05
18	EIF3A_cg08078028_	< 0.01	0.05
19	CXXC5_cg14871225_	< 0.001	0.06
20	UHRF1_cg18351781_	< 0.01	0.06
21	DEF8_cg05185784_	< 0.05	0.06
22	SLX4_cg08367804_	< 0.0001	0.1

We also used the CTD database to analyze relationships between the above genes and diseases. Table 5 showed that the genes were related to immune system diseases. All the 5 genes were related to inflammation. In addition, PIK3CD and SPI1 were also related to different types of lymphoma.

**Table 5:** Relationship to immune system diseases related to CpG sites in the validation assay based on the CTD database

Gene	Disease
PIK3CD	Lymphoma, Large B-Cell, Diffuse^a^; Immunodeficiency^a^;Lymphoma, Mantle-Cell^a^; Inflammation
CXXC5	Inflammation
LTBR	Malaria^a^; Inflammation
SPI1	Leukemia, Myeloid, Acute^a^; Precursor T-Cell Lymphoblastic Leukemia-Lymphoma^a^; Inflammation
SERPINB9	Inflammation

^a^Direct evidence of marker or mechanism in this disease in the CTD database

## Discussion

Our study showed that infants born by CDMR had more locus-specific DNA hypermethylation in genes that were related to the immune system. However, the differences might be largely explained by the cell type proportions.

Evidence has indicated that CD may be associated with the development of various disorders in childhood. Short-term risks of CD include altered immune development, an increased likelihood of allergy, atopy, and asthma, and reduced intestinal gut microbiome diversity [20]. At 6 months of age, children born by CD had a significantly higher BMI [21]. Continuously, CD was associated with offspring obesity until age 20 to 28 years [8]. Besides, CD was weakly associated with otitis media in childhood, especially in the first 7 years of life [22]. In China, children born by CDMR prior to 39 gestational weeks were identified with an increased risk of emotional and behavioral problems at preschool age [23]. Moreover, emergency or planned CD was consistently associated with a modestly increased risk of autism spectrum disorder (ASD) from gestational weeks of 36 to 42 when compared with vaginal delivery in four Nordic countries and Western Australia [24].

Epigenetic changes have been shown to be essential during hematopoiesis by contributing to the formation of stable, heritable gene expression patterns. Aberrant DNA methylation has been associated with several immune deficiencies and autoimmune disorders [25]. A number of studies have added to the growing body of evidence supporting significant associations of epigenetic regulation of gene expression and asthma and allergy phenotypes [26]. DNA hypermethylation has also been identified to be related to more than six types of autoimmune diseases, including rheumatoid arthritis, Sjogren’s syndrome, Graves’ disease, Type 1 diabetes, and Crohn’s disease [27].

A previous study found that DNA-methylation in fetal leucocytes was higher in CDMR births than that in VD [9]. It was also reported that methylation of single genes (e.g., *ELA2* and *IRF1*) was significantly elevated in elective CD than in VD [10]. These results were consistent with our findings. Our study extended and refined evidence in this area. After the GO enrichment, we found 20 GO terms, most of which were related to the immune system. In the present pathway analysis, the DMPs were overlapped with 10 KEGG pathways, including asthma and several autoimmunity diseases.

Moreover, several genes involved in the normal function of the immune system were differentially methylated in VD comparing to CDMR. Based on the validation analysis, the different methylation status was also found in PIK3CD, CXXC5, LTBR, SPI1 and SERPINB9 genes. Of them, three genes were involved in the maturation and normal function of B cells, including PIK3CD, CXXC5 and SPI1. The PIK3CD, one of the phosphoinositide 3-kinase (PI3K) catalytic subunits, has emerged as a critical mediator of multiple B cell functions [28]. PIK3CD was confirmed to participate in the proliferation of B cells and activation in humoral immunity, mediating the survival pathways of T cells and B cells [29]. Based on the previous study, CXXC5 was also involved in the regulation of Toll-like receptors (TLRs) response and antibody production in B cells [30]. Additionally, SPI1 encoded an ETS-domain transcription factor that activated gene expression during myeloid and B-lymphoid cell development [31].

The other 2 genes participated in the process of immune response. LTBR was one of the subunits of lymphotoxin, which was a member of the tumor necrosis factor (TNF) superfamily [32]. The LTBR activation induced the NFκB pathways, which leads to inflammatory gene transcription and nuclear translocation, had a major role in lymphoid organogenesis, immune response and initiation of inflammation [33, 34]. Moreover, SERPINB9 was considered as the main inhibitor for the cytotoxic effector protein granzyme B and caspase-1, which was involved in the immune response [35, 36].

These changes in the above gene expression levels had profound implications for immune function development in the neonates. The immunity abnormity could lead to a series of chronic inflammation diseases and autoimmune diseases, including viral encephalitis, hepatitis B, IgA nephropathy, type 1 diabetes, lymphoblastic leukemia, and cancers [30, 37–40].

However, the mechanisms on how different delivery mode affects different DNA methylation status remained unclear. A plausible explanation was that the long and extreme fetal stress during labor and his physiological responses might program DNA methylation and gene expression [10]. It was also reported that fetal exposure to glucocorticoids may change the epigenetic state, affecting both DNA methylation and H3K9 acetylation, resulting in significant variations in genome-wide promoter methylation [41]. Consequently, the mode of delivery may affect the banking quality in terms of the number of fetal cells [42].

We also observed that cell type proportions in the umbilical cord blood of CDMR and VD infants explained a large portion of variation in analyses of DNA methylation patterns. When we selected DMPs on M-value, the number of DMPs showing significant DNA methylation associated with delivery mode was reduced from 1208 to 2 single CpG when controlling for blood cell type proportions. After the estimation of cell types in cord blood using FlowSorted.CordBloodCombined.450 k, significant differences were found in CD8T cells, CD4T cells, B cells and Granulocytes between the two groups. More CD8T cells, CD4T cells and B cells were found in CDMR group, while more Granulocytes were found in VD group. It was reported that neonates born by caesarean delivery had lower T and NK lymphocyte counts than neonates born by vaginal delivery. Within the T-lymphocyte population, helper and naive T lymphocyte counts decreased in neonates born by caesarean delivery [43]. Significant associations were also found between basophilic granulocyte counts, plasmacytoid dendritic cells and delivery mode [44].

The present study used cord blood, which were mixed-cell samples. The previous studies using blood samples from both adults and newborns illustrated that a large number of DMPs could be influenced by the cellular composition of the blood sample [45, 46]. Similar results were also found in gene expression study that used adult blood samples to define the cell type specific gene expression patterns [47]. Purification of the specific cell type would be the most reliable approach for eliminating the interindividual variability. However, a large volume of blood and specialized technology would be required to obtain sufficient number of various cell types.

Our study has several strengths. First, only women who had CDMR were selected as the exposed group in our study, to avoid any potential effects of maternal and/or fetal complications. This study is the first to examine the effect of CDMR on DNA methylation status in the umbilical cord blood of the infants. Second, the cord blood DNA methylation status was measured using commercial DNA methylation chip and targeted bisulfite sequencing assay, which provided reliable assessments. Third, the women who had CDMR and VD were matched on the basic characteristics, to avoid the potential confounding effects.

Nevertheless, our study had limitations. The sample size was still small, which may have limited our ability to identify more differentially methylated positions. In addition, there may be many crucial DMPs not included in the commercial chip. Finally, the cell proportions in cord blood were not well considered in the present analysis as our stored samples were no longer suitable for cell typing. Although we have used FlowSorted.CordBloodCombined.450 k to estimate the proportion of cells, further methylation analysis in specific cell types would be more reliable. Further studies focusing on the specific pathway with larger sample size should be performed.

## Conclusions

Our study suggested that DNA methylation patterns were altered in different delivery modes, while the differences might be largely explained by the cell type proportions. Further studies investigating the DNA methylation status of the specific genes may be essential to understand the long-lasting effect of the delivery mode on the offspring’s health.

## Supplementary Information

**Additional file 1 Table S1**. Information of primers used in targeted bisulfite sequencing assay. **Table S2.** Differentially methylated loci between vaginal delivery and CDMR group from the Methylation Chip. **Table S3.** Differentially methylated loci between vaginal delivery and CDMR group from targeted bisulfite sequencing assay. **Table S4**. The comparison of M-values between VD and CDMR group using multiple linear regression. **Table S5**. The estimate proportions of cell type in cord blood. **Table S6**. Differentially methylated CpG sites between VD and CDMR group from the Methylation Chip analyzed by M values, under the threshold difference in the mean β value |Δβ| ≥ 10%. **Fig. S1**. A volcano plot of lg-transformed *P* values vs differences in DNA methylation (b-value) between VD and CDMR group.

## Data Availability

The datasets used and analyzed during the current study are available from the corresponding author on reasonable request.

## Abbreviations

CDMR: Cesarean deliveries on maternal request
VD: Vaginal deliveries
DMPs: Differentially methylated positions
CD: Cesarean delivery
BMI: Body mass index
CpG: Cytosine phosphate-guanine
GO: Gene ontology
KEGG: Kyoto Encyclopedia of Genes and Genomes
HLA: Human leukocyte antigen
PI3K: Phosphoinositide 3-kinase
TCRs: T cell antigen receptors
BCRs: B cell antigen receptors
TLRs: Toll-like receptors
LTBR: Lymphotoxin β receptor
TNF: Tumor necrosis factor
SERPINB9: Serine protease inhibitor B9
