# Supplementary material for: The impact of cesarean delivery on infant DNA methylation
Source: BMC Pregnancy Childbirth. 2021 Mar 30;21:265. doi: 10.1186/s12884-021-03748-y (PMC8011183; doi:10.1186/s12884-021-03748-y)
Supplement: Supplementary file 1 — Additional file 1 Table S1. Information of primers used in targeted bisulfite sequencing assay. Table S2. Differentially methylated loci between vaginal delivery and CDMR group from the Methylation Chip. Table S3. Differentially methylated loci between vaginal delivery and CDMR group from targeted bisulfite sequencing assay. Table S4. The comparison of M-values between VD and CDMR group using multiple linear regression. Table S5. The estimate proportions of cell type in cord blood. Table S6. Differentially methylated CpG sites between VD and CDMR group from the Methylation Chip analyzed by M values, under the threshold difference in the mean β value |Δβ| ≥ 10%. Fig. S1. A volcano plot of lg-transformed P values vs differences in DNA methylation (b-value) between VD and CDMR group. [file 12884_2021_3748_MOESM1_ESM.zip › Supplemental Table- The impact of cesarean delivery on infant DNA methylation0319.docx]

**Supplemental Material**

**The impact of cesarean delivery on infant DNA methylation**

Qian Chen^1^, Yanhong Ming^1^, Yuexin Gan^1^, Lisu Huang^1,2^, Yanjun Zhao^1,3^, Xia Wang^1^, Yongjie Liu^1^, Jun Zhang^1,^ *

^1^Ministry of Education-Shanghai Key Laboratory of Children's Environmental Health, Xinhua Hospital, Shanghai Jiao Tong University School of Medicine, Shanghai 200092, China.

^2^Department of Obstetrics and Gynecology, Hospital of Obstetrics and Gynecology, Fudan University, Shanghai 200011, China.

^3^Department of Pediatrics, Xinhua Hospital, Shanghai Jiao Tong University School of Medicine, Shanghai 200092, China.

^4^Department of Child Health Care, Shanghai Children’s Hospital, Shanghai Jiao Tong University, Shanghai 200040, China.

*Correspondence and requests for materials should be addressed to J Z (zhangjun@xinhuamed.com.cn. Xinhua Hospital, Shanghai Jiao Tong University School of Medicine, 1665 Kongjiang Road, Shanghai 200092, China)

**Table of contents**

**Table S1**. Information of primers used in targeted bisulfite sequencing assay

**Table S2.** Differentially methylated loci between vaginal delivery and CDMR group from the Methylation Chip

**Table S3.** Differentially methylated loci between vaginal delivery and CDMR group from targeted bisulfite sequencing assay

**Table S4**. The comparison of M-values between VD and CDMR group using multiple linear regression

**Table S5**. The estimate proportions of cell type in cord blood

**Table S6**. Differentially methylated CpG sites between VD and CDMR group from the Methylation Chip analyzed by M values, under the threshold difference in the mean β value |Δβ| ≥ 10%

**Caption of Figures: Figure S1**. A volcano plot of lg-transformed P values vs differences in DNA methylation (b-value) between VD and CDMR group.

| Table S1. Information of primers used in targeted bisulfite sequencing assay | | |
| --- | --- | --- |
| Order | Primer Name | Seqence |
| 1 | ADGRA2_cg12869334_F | TTTGTAGTTGGTTTAGAGTTAGGTGTG |
| 2 | ADGRA2_cg12869334_R | CCTTAAACCRATTCTTACCRCAAAC |
| 3 | AGTRAP_cg25467652_F | ATAAAACACAAACATAAAACACAAAC |
| 4 | AGTRAP_cg25467652_R | TGGYGAAAGTTGGAGTTTTTGT |
| 5 | BAHCC1_cg14170999_F | GTGGGTTGTTTTTGTTTGTTGT |
| 6 | BAHCC1_cg14170999_R | CCRACCTCATAACCAAAACCTAAA |
| 7 | CDH22_cg07296387_F | GTTTYGGGYGGGGGTTTT |
| 8 | CDH22_cg07296387_R | CCRTAATCCTAAAAACCCTCAACAA |
| 9 | CPT1B_cg24363820_F | CCTAAAAATAAAACCRACCTAAACAAA |
| 10 | CPT1B_cg24363820_R | YGATTTGGGAGTAGGTGTTAGGT |
| 11 | CXXC5_cg14871225_F | AAACAACCTCCCCCACCTC |
| 12 | CXXC5_cg14871225_R | GTTGGGGATGGGGTGATT |
| 13 | CXXC5_cg19628988_F | CCCAACAACCCCRAAAAAC |
| 14 | CXXC5_cg19628988_R | TGTTAGAGGTTGGGTTGTGGT |
| 15 | CXXC5_cg20455854_F | AACCTCCCCTTTTCAAAAACCT |
| 16 | CXXC5_cg20455854_R | TTTTAAGTGTGTGTTTGGGTGTGT |
| 17 | DEF8_cg05185784_F | TGGGTAGATAGGAYGTTGTTGATTT |
| 18 | DEF8_cg05185784_R | ACCCTAAACCRAAACTCAAAACA |
| 19 | DENND3_cg14480530_F | CCCACTAAACCACAACATCCTC |
| 20 | DENND3_cg14480530_R | GGTTTTGTAGGGGTGAGTTGAG |
| 21 | DGKA_cg07679948_F | GGGGTTAGGGATTTTGTTAGAGTT |
| 22 | DGKA_cg07679948_R | CTTCCTAACCATAACCCCATTTT |
| 23 | DOC2B_cg14984781_F | GTGTGTAGAGATAYGTGGTGGAAGT |
| 24 | DOC2B_cg14984781_R | CCCAAAATCCCCTAATCACTTCT |
| 25 | EIF3A_cg08078028_F | TCTTAATCCTCTATCCTCATCAACTATTC |
| 26 | EIF3A_cg08078028_R | GGTATGGATGATGATAGAGGTTTTAG |
| 27 | ENOSF1_cg07100532_F | AGGGAGAAGTYGGGTTTGAG |
| 28 | ENOSF1_cg07100532_R | ACATCAATACRAATATAATCAATAATAACCTACCTC |
| 29 | HSPG2_cg21272897_F | GGGGTTTGTTTGAGGGTTTY |
| 30 | HSPG2_cg21272897_R | ACCCRATAAACAACCRACCACAC |
| 31 | LDLRAD4_cg04967578_F | CAAAAACCRCTCAACCCCTCT |
| 32 | LDLRAD4_cg04967578_R | GAGAYGAAGGGTGGGTTTAGG |
| 33 | LOC441666_cg06833823_F | TACATCCACAAATCCTACTCACC |
| 34 | LOC441666_cg06833823_R | GTTGGATTGGAAGTTGGGATTT |
| 35 | LOC441666_cg23026554_F | TTACATCCACAAATCCTACTCACC |
| 36 | LOC441666_cg23026554_R | GTTGGATTGGAAGTTGGGATTT |
| 37 | LOC441666_cg27119456_F | CCRAAATCCCAACTTCCAATC |
| 38 | LOC441666_cg27119456_R | TGTTTTTATAGGTTTAGTTGTTGTGGT |
| 39 | LTBR_cg19476647_F | GGATTGGGTTGGGTTGGTAG |
| 40 | LTBR_cg19476647_R | AAACCCAAAACRAACAATCATAAAA |
| 41 | MAG_cg22238209_F | AGGGGAAATTGGAGGGTTTT |
| 42 | MAG_cg22238209_R | CTTCACCACRCACAAACACTACAC |
| 43 | MOBP_cg11235602_F | AAAAACTAAATCTACTACRCCTACCAAAAA |
| 44 | MOBP_cg11235602_R | TAGGAGTTGGAAATAGGTAGGAAGGT |
| 45 | MYL9_cg18660345_F | GTAGGGGTTTTTGGGAGTTTT |
| 46 | MYL9_cg18660345_R | ACCCCTTCCCCTCCAAAA |
| 47 | NCAPH2_cg25152348_F | GGAATAGTAAAATGGYGTTAGAATTAGTGG |
| 48 | NCAPH2_cg25152348_R | CCCTTACCTCCTCCAAATACTC |
| 49 | NCLN_cg09462281_F | GTTTTTGGGGTGGTTTATTGAG |
| 50 | NCLN_cg09462281_R | TTCACCCCTACAAATCTCAACA |
| 51 | NPAS3_cg01993818_F | CTACCCTTCCCCRTCTACAACA |
| 52 | NPAS3_cg01993818_R | TGAAAGAAAGAGGGGGTGGA |
| 53 | PAX8_cg07594247_F | GAAGGGTGAGTGAGGATTTGT |
| 54 | PAX8_cg07594247_R | TTCTCTCTCCCCRCCTTACC |
| 55 | PIK3CD_cg23098018_F | TTCACCTACATCAACCAAACAAC |
| 56 | PIK3CD_cg23098018_R | GGTGGGGTAGGTAGGGTTAGTT |
| 57 | PON1_cg17330251_F | GGTTAGGAGGTTTTGTTTGTTTGT |
| 58 | PON1_cg17330251_R | TCCCCTCCCCRACTAAACTAAA |
| 59 | PON1_cg19678392_F | TATTGGTAAGAAGATTGGTGGTTTT |
| 60 | PON1_cg19678392_R | AAACCACRCCTTCTATACACCTAATC |
| 61 | PRKAR1B_cg11064039_F | CCTTCTCCAAACCCCCTAAA |
| 62 | PRKAR1B_cg11064039_R | AGTTTGTTTGTTGGGTTTTGTTTT |
| 63 | PRTN3_cg19787694_F | GTTTTGAGGTGGTGGGTGT |
| 64 | PRTN3_cg19787694_R | CAACCCATAACCAAACACTAAATACC |
| 65 | RASA3_cg21261158_F | CCCCCAAAACACCCTTAAAAC |
| 66 | RASA3_cg21261158_R | AGGGTTTGGTTTTGAGGTTTGT |
| 67 | RCAN3_cg25140783_F | GGTTGTTTTGGTGGGTTTTG |
| 68 | RCAN3_cg25140783_R | AAAACAAATCAAATCAACTACTACTCTACAC |
| 69 | SERPINB9_cg01345354_cg10863922_F | TTCTACCCRCAAAAATATCCCTAAA |
| 70 | SERPINB9_cg01345354_cg10863922_R | TTAGGAGGAGTAAAGGTTAGTGTAGATG |
| 71 | SIAH2_cg24856383_F | CACRACRCCCAACCCAAATC |
| 72 | SIAH2_cg24856383_R | TTTAGAAGYGGGTGGGTTAGG |
| 73 | SLC12A7_cg18997983_F | TAGGAGGGAYGGGTAGGTTATTG |
| 74 | SLC12A7_cg18997983_R | CCCACCCTCAACTAAAACTCTC |
| 75 | SLX4_cg08367804_F | GAGGGGTTTTTGGAGGTTTTT |
| 76 | SLX4_cg08367804_R | ACCCCCTAAACACCAACAAC |
| 77 | SPI1_cg15982099_F | ACTTCCCACTAATAACAAACCAAAA |
| 78 | SPI1_cg15982099_R | TGAGGGGTTTTGTATTGGTTTTT |
| 79 | TSPOAP1_cg17525495_F | AGTTAGATTTTTAAGTTTTGGGGGTTTT |
| 80 | TSPOAP1_cg17525495_R | ACTAACCAAACCTAACCCCAACTAC |
| 81 | TUBGCP6_cg19851816_F | TCAACTACACAACAATCTCATCAAC |
| 82 | TUBGCP6_cg19851816_R | GTTTTGGGTTGGTTGGTTTTT |
| 83 | UHRF1_cg18351781_F | GGGGTTGTTTTTGATGGAGTT |
| 84 | UHRF1_cg18351781_R | CCATCCCAAACACACCTACA |
| 85 | ZBTB7A_cg16242615_F | CRTTCCCCTCCTCCAAAA |
| 86 | ZBTB7A_cg16242615_R | GGGGTTTGTTAGGGTTTGTTTTT |
| 87 | ZFP36L1_cg08169020_F | GGGGTGGYGGGTTAGGTT |
| 88 | ZFP36L1_cg08169020_R | CAACTCCTCAACAACCTCAAAAA |
| 89 | ZFP36L1_cg10099732_F | GGTTGTTTTTGGGTGGGTAG |
| 90 | ZFP36L1_cg10099732_R | TCAATCACCCTACCCAACTCC |

| Table S2. Differentially methylated CpG sites between vaginal delivery and CDMR group from the Methylation Chip | | | | |
| --- | --- | --- | --- | --- |
| TargetID | MethylDiff | CHR | MAPINFO | UCSC_REFGENE_NAME |
| cg21211688 | 0.25 | 9 | 136403935 | ADAMTSL2 |
| cg11495604 | 0.22 | 20 | 62053198 | KCNQ2 |
| cg11251367 | 0.21 | 1 | 240620177 | FMN2 |
| cg10277872 | 0.21 | 4 | 4136918 |  |
| cg08514194 | 0.20 | 21 | 46075092 | KRTAP12-4; C21orf29 |
| cg22543924 | 0.19 | 12 | 9065171 |  |
| cg12303247 | 0.19 | 1 | 155853542 | SYT11 |
| cg15161973 | 0.19 | 10 | 132909153 | TCERG1L |
| cg00540941 | 0.19 | 6 | 32974843 | HLA-DOA |
| cg11585022 | 0.18 | 5 | 110427346 | WDR36 |
| cg18486102 | 0.17 | 12 | 50297777 | FAIM2 |
| cg11019305 | 0.17 | 5 | 171708886 | UBTD2 |
| cg13488137 | 0.17 | 10 | 134956732 |  |
| cg11882358 | 0.17 | 3 | 101658926 | LOC152225 |
| cg11844537 | 0.16 | 10 | 132911103 | TCERG1L |
| cg05362314 | 0.16 | 4 | 4137166 |  |
| cg19653246 | 0.16 | 14 | 101505898 | MIR654; MIR376B; MIR376A1; MIR376A2; MIR376C |
| cg08617581 | 0.16 | 10 | 132911152 | TCERG1L |
| cg26465155 | 0.16 | 11 | 35611044 |  |
| cg16248432 | 0.16 | 12 | 50974843 | DIP2B |
| cg25649515 | 0.16 | 3 | 177309023 |  |
| cg09867002 | 0.15 | 10 | 1416776 | ADARB2 |
| cg27468880 | 0.15 | 7 | 965995 | ADAP1 |
| cg10738003 | 0.15 | 11 | 120233535 | ARHGEF12 |
| cg09157251 | 0.15 | 11 | 70733251 | SHANK2 |
| cg11872966 | 0.15 | 10 | 132910868 | TCERG1L |
| cg23130832 | 0.15 | 10 | 132909289 | TCERG1L |
| cg15029183 | 0.15 | 6 | 143586006 | AIG1 |
| cg06063541 | 0.15 | 10 | 132909113 | TCERG1L |
| cg27433479 | 0.15 | 1 | 1687481 | NADK |
| cg15567368 | 0.15 | 7 | 563891 |  |
| cg19236675 | 0.15 | 7 | 76624761 | PMS2L11 |
| cg17839758 | 0.15 | 21 | 46077562 | C21orf29; KRTAP12-3 |
| cg07227024 | 0.14 | 2 | 202163482 | ALS2CR12 |
| cg13573375 | 0.14 | 19 | 4033718 | PIAS4 |
| cg00051154 | 0.13 | 2 | 237079099 |  |
| cg08786370 | 0.13 | 4 | 8527460 |  |
| cg10911276 | 0.13 | 4 | 4142417 |  |
| cg04628741 | 0.13 | 10 | 18425598 |  |
| cg06732825 | 0.13 | 12 | 132916669 |  |
| cg07584620 | 0.13 | 1 | 2265881 | MORN1 |
| cg13854498 | 0.13 | 10 | 132600304 |  |
| cg25674027 | 0.13 | 12 | 103325781 |  |
| cg02741327 | 0.12 | 2 | 135214041 | TMEM163 |
| cg07748255 | 0.12 | 4 | 1324877 | MAEA |
| cg15460035 | 0.12 | 12 | 132929908 |  |
| cg27346510 | 0.12 | 10 | 132911037 | TCERG1L |
| cg22741595 | 0.12 | 11 | 77532707 | C11orf67; RSF1 |
| cg11680857 | 0.12 | 1 | 152635200 | LCE2D |
| cg24739935 | 0.12 | 12 | 79814928 | SYT1 |
| cg04368836 | 0.12 | 17 | 2263865 | SGSM2 |
| cg11987751 | 0.12 | 1 | 1663860 | SLC35E2 |
| cg24137123 | 0.11 | 5 | 178404926 |  |
| cg13066461 | 0.11 | 11 | 19078052 | MRGPRX2 |
| cg22931151 | 0.11 | 5 | 180581761 | OR2V2 |
| cg19556341 | 0.11 | 4 | 4167577 |  |
| cg17759595 | 0.11 | 8 | 26481696 | DPYSL2 |
| cg27244972 | 0.11 | 21 | 47716529 | C21orf57 |
| cg24441810 | 0.11 | 2 | 120436039 | TMEM177 |
| cg26337497 | 0.10 | 3 | 31935279 | OSBPL10 |
| cg11573390 | 0.10 | 10 | 132909470 | TCERG1L |
| cg25830305 | 0.10 | 11 | 1859381 | TNNI2 |
| cg23825480 | 0.10 | 22 | 31336785 | MORC2 |
| cg22804805 | 0.10 | 7 | 150765472 | SLC4A2 |
| cg16261114 | -0.10 | 15 | 99551997 | LOC145814 |
| cg13641645 | -0.10 | 9 | 6932042 | KDM4C |
| cg04311686 | -0.10 | 5 | 1179328 |  |
| cg17009574 | -0.10 | 6 | 26365345 | BTN3A2 |
| cg02952913 | -0.10 | 1 | 247336686 | ZNF124 |
| cg01256139 | -0.10 | 2 | 161228737 | RBMS1 |
| cg06334689 | -0.10 | 16 | 30018720 | DOC2A |
| cg07837085 | -0.10 | 1 | 160708990 | SLAMF7 |
| cg15293181 | -0.10 | 2 | 1166311 | SNTG2 |
| cg19816075 | -0.10 | 12 | 113408421 | OAS3 |
| cg04824555 | -0.11 | 9 | 141105974 | FAM157B |
| cg23098789 | -0.11 | 10 | 134920382 | GPR123 |
| cg01471923 | -0.11 | 20 | 23015091 | SSTR4 |
| cg03024489 | -0.11 | 2 | 87040878 |  |
| cg22238209 | -0.11 | 19 | 35800743 | MAG |
| cg20089799 | -0.11 | 12 | 3384898 | TSPAN9 |
| cg13111532 | -0.11 | 1 | 1886543 | KIAA1751 |
| cg13740636 | -0.11 | 11 | 93754223 | HEPHL1 |
| cg21700663 | -0.11 | 15 | 101093900 |  |
| cg08158105 | -0.11 | 12 | 125509502 | BRI3BP |
| cg22836174 | -0.11 | 2 | 228231970 | TM4SF20 |
| cg13595143 | -0.11 | 9 | 472966 |  |
| cg05008296 | -0.11 | 14 | 68163792 | RDH11 |
| cg23159165 | -0.11 | 11 | 107567020 |  |
| cg06546677 | -0.11 | 8 | 145537504 | HSF1 |
| cg13183651 | -0.12 | 17 | 39151857 | KRTAP3-3 |
| cg20848488 | -0.12 | 4 | 6417932 | PPP2R2C |
| cg14279361 | -0.12 | 19 | 6721955 | C3 |
| cg10680514 | -0.12 | 19 | 40093111 | LGALS13 |
| cg22681945 | -0.12 | 1 | 37732046 |  |
| cg22373097 | -0.12 | 21 | 32129060 | KRTAP21-1 |
| cg24844518 | -0.12 | 5 | 156811669 | CYFIP2 |
| cg22971153 | -0.12 | 2 | 113825302 | IL1F10 |
| cg04497611 | -0.12 | 17 | 80860250 | TBCD |
| cg22112152 | -0.12 | 3 | 153501841 |  |
| cg07743747 | -0.12 | 3 | 46394550 | CCR2 |
| cg12475092 | -0.12 | 5 | 84934437 |  |
| cg17813879 | -0.12 | 8 | 19252188 | SH2D4A |
| cg23333490 | -0.12 | 6 | 33050741 | HLA-DPB1 |
| cg06924902 | -0.12 | 8 | 135609134 | ZFAT; ZFATAS |
| cg20370184 | -0.13 | 6 | 31838544 | SLC44A4 |
| cg15971518 | -0.13 | 11 | 57159174 | PRG2 |
| cg01823585 | -0.13 | 7 | 1018969 |  |
| cg01412404 | -0.13 | 3 | 14289162 |  |
| cg08912652 | -0.13 | 11 | 130779479 | SNX19 |
| cg18949721 | -0.13 | 14 | 99858181 |  |
| cg15837943 | -0.13 | 2 | 231734413 | ITM2C |
| cg25049941 | -0.13 | 6 | 32728953 | HLA-DQB2 |
| cg09462281 | -0.13 | 19 | 3191030 | NCLN |
| cg15226348 | -0.13 | 5 | 7540955 | ADCY2 |
| cg11768182 | -0.13 | 19 | 691833 | PRSSL1 |
| cg17628491 | -0.13 | 17 | 78867822 | RPTOR |
| cg21974656 | -0.13 | 11 | 44649802 |  |
| cg09854620 | -0.13 | 10 | 131930596 |  |
| cg21932934 | -0.13 | 11 | 120039306 |  |
| cg11723923 | -0.13 | 13 | 112820997 |  |
| cg14341177 | -0.13 | 9 | 95475787 | BICD2 |
| cg08506672 | -0.13 | 5 | 3959743 |  |
| cg11144986 | -0.13 | 12 | 132935765 |  |
| cg07889790 | -0.14 | 8 | 622078 | ERICH1 |
| cg23681001 | -0.14 | 1 | 53936382 |  |
| cg14195178 | -0.14 | 17 | 80849463 | TBCD |
| cg08102564 | -0.14 | 17 | 19620263 | SLC47A2 |
| cg19591135 | -0.14 | 7 | 905795 | UNC84A |
| cg06499415 | -0.14 | 9 | 141042435 |  |
| cg13653328 | -0.15 | 5 | 148520669 | ABLIM3 |
| cg21272897 | -0.15 | 1 | 22191453 | HSPG2 |
| cg22933800 | -0.15 | 6 | 32605704 | HLA-DQA1 |
| cg15135990 | -0.15 | 2 | 74665089 | RTKN |
| cg12031275 | -0.15 | 15 | 101093834 |  |
| cg08629394 | -0.16 | 12 | 96188933 |  |
| cg11897887 | -0.16 | 20 | 23550632 | CST9L |
| cg14494781 | -0.16 | 6 | 2615341 |  |
| cg11400162 | -0.16 | 6 | 170455498 |  |
| cg27577781 | -0.17 | 2 | 235953159 | SH3BP4 |
| cg13064658 | -0.17 | 1 | 212003989 | LPGAT1 |
| cg03122926 | -0.17 | 7 | 2425741 |  |
| cg06293782 | -0.17 | 6 | 32713188 | HLA-DQA2 |
| cg04246708 | -0.17 | 1 | 246785611 | CNST |
| cg07240846 | -0.18 | 10 | 12438782 | CAMK1D |
| cg02444957 | -0.18 | 2 | 69440951 | ANTXR1 |
| cg10920758 | -0.18 | 10 | 134974555 | KNDC1 |
| cg04777551 | -0.18 | 6 | 32628953 | HLA-DQB1 |
| cg16886051 | -0.18 | 11 | 6744442 | GVIN1 |
| cg17373649 | -0.18 | 8 | 669578 | ERICH1 |
| cg21388339 | -0.18 | 1 | 3606550 | TP73 |
| cg05176970 | -0.19 | 17 | 724273 | NXN |
| cg13295089 | -0.19 | 7 | 155492281 | RBM33 |
| cg02902672 | -0.19 | 6 | 32635360 | HLA-DQB1 |
| cg05023192 | -0.19 | 2 | 240965916 | NDUFA10 |
| cg00616572 | -0.19 | 16 | 10772249 | TEKT5 |
| cg12474444 | -0.20 | 5 | 1298644 |  |
| cg21442271 | -0.20 | 22 | 18738315 |  |
| cg05308244 | -0.21 | 12 | 130968528 | RIMBP2 |
| cg14799809 | -0.21 | 6 | 32608095 | HLA-DQA1 |
| cg24629711 | -0.21 | 3 | 52869263 | MUSTN1 |
| cg11784298 | -0.22 | 6 | 32610971 | HLA-DQA1 |
| cg18500967 | -0.22 | 7 | 17826367 |  |
| cg16471877 | -0.23 | 6 | 99817927 | COQ3 |
| cg10528424 | -0.24 | 11 | 1858572 | SYT8 |
| cg02201753 | -0.29 | 10 | 135052900 | VENTX |

MethylDiff: the difference between vaginal delivery and CDMR group in each locus

CHR: Chromosome

MAPINFO: Coordinate information

UCSC_REFGENE_NAME: UCSC Reference Gene Name

| Table S3. Differentially methylated CpG sites between vaginal delivery and CDMR group from targeted bisulfite sequencing assay | | |
| --- | --- | --- |
| Target | MethylDiff | P |
| SLX4_cg08367804_ | -0.14 | 0.00 |
| SLX4_cg08367804_ | -0.13 | 0.00 |
| SLX4_cg08367804_ | -0.13 | 0.00 |
| SLX4_cg08367804_ | -0.13 | 0.00 |
| SLX4_cg08367804_ | -0.12 | 0.00 |
| SLX4_cg08367804_ | -0.12 | 0.00 |
| SLX4_cg08367804_ | -0.12 | 0.00 |
| SLX4_cg08367804_ | -0.11 | 0.00 |
| SLX4_cg08367804_ | -0.11 | 0.00 |
| SLX4_cg08367804_ | -0.11 | 0.00 |
| SLX4_cg08367804_ | -0.11 | 0.00 |
| SLX4_cg08367804_ | -0.11 | 0.00 |
| SLX4_cg08367804_ | -0.11 | 0.00 |
| SLX4_cg08367804_ | -0.11 | 0.00 |
| SLX4_cg08367804_ | -0.11 | 0.00 |
| TUBGCP6_cg19851816_ | -0.10 | 0.00 |
| DEF8_cg05185784_ | -0.09 | 0.02 |
| CXXC5_cg14871225_ | -0.08 | 0.00 |
| DEF8_cg05185784_ | -0.08 | 0.03 |
| DEF8_cg05185784_ | -0.08 | 0.03 |
| DEF8_cg05185784_ | -0.08 | 0.01 |
| EIF3A_cg08078028_ | -0.08 | 0.00 |
| DEF8_cg05185784_ | -0.08 | 0.02 |
| CXXC5_cg14871225_ | -0.08 | 0.00 |
| EIF3A_cg08078028_ | -0.08 | 0.00 |
| EIF3A_cg08078028_ | -0.07 | 0.00 |
| UHRF1_cg18351781_ | -0.07 | 0.00 |
| CXXC5_cg14871225_ | -0.07 | 0.00 |
| CXXC5_cg14871225_ | -0.07 | 0.00 |
| AGTRAP_cg25467652_ | -0.07 | 0.00 |
| UHRF1_cg18351781_ | -0.07 | 0.00 |
| CXXC5_cg14871225_ | -0.07 | 0.00 |
| UHRF1_cg18351781_ | -0.07 | 0.00 |
| UHRF1_cg18351781_ | -0.07 | 0.00 |
| CXXC5_cg14871225_ | -0.07 | 0.00 |
| UHRF1_cg18351781_ | -0.07 | 0.00 |
| UHRF1_cg18351781_ | -0.07 | 0.00 |
| UHRF1_cg18351781_ | -0.07 | 0.00 |
| UHRF1_cg18351781_ | -0.07 | 0.00 |
| DEF8_cg05185784_ | -0.07 | 0.04 |
| PRTN3_cg19787694_ | -0.07 | 0.00 |
| EIF3A_cg08078028_ | -0.07 | 0.00 |
| PRTN3_cg19787694_ | -0.07 | 0.00 |
| UHRF1_cg18351781_ | -0.07 | 0.01 |
| CXXC5_cg14871225_ | -0.07 | 0.00 |
| PRTN3_cg19787694_ | -0.07 | 0.00 |
| AGTRAP_cg25467652_ | -0.07 | 0.00 |
| EIF3A_cg08078028_ | -0.07 | 0.00 |
| CXXC5_cg14871225_ | -0.07 | 0.00 |
| PRTN3_cg19787694_ | -0.06 | 0.00 |
| EIF3A_cg08078028_ | -0.06 | 0.00 |
| UHRF1_cg18351781_ | -0.06 | 0.01 |
| PRTN3_cg19787694_ | -0.06 | 0.00 |
| NCLN_cg09462281_ | -0.06 | 0.00 |
| EIF3A_cg08078028_ | -0.06 | 0.00 |
| AGTRAP_cg25467652_ | -0.06 | 0.00 |
| TUBGCP6_cg19851816_ | -0.06 | 0.00 |
| CXXC5_cg14871225_ | -0.06 | 0.02 |
| TUBGCP6_cg19851816_ | -0.06 | 0.00 |
| CXXC5_cg14871225_ | -0.06 | 0.00 |
| PRTN3_cg19787694_ | -0.06 | 0.00 |
| PRTN3_cg19787694_ | -0.06 | 0.00 |
| CXXC5_cg14871225_ | -0.06 | 0.02 |
| CXXC5_cg14871225_ | -0.06 | 0.01 |
| TSPOAP1_cg17525495_ | -0.06 | 0.00 |
| SLC12A7_cg18997983_ | -0.05 | 0.00 |
| SLC12A7_cg18997983_ | -0.05 | 0.00 |
| DEF8_cg05185784_ | -0.05 | 0.00 |
| EIF3A_cg08078028_ | -0.05 | 0.01 |
| TUBGCP6_cg19851816_ | -0.05 | 0.00 |
| SPI1_cg15982099_ | -0.05 | 0.00 |
| DEF8_cg05185784_ | -0.05 | 0.00 |
| SLC12A7_cg18997983_ | -0.05 | 0.00 |
| AGTRAP_cg25467652_ | -0.05 | 0.00 |
| LTBR_cg19476647_ | -0.05 | 0.00 |
| NCLN_cg09462281_ | -0.05 | 0.00 |
| SPI1_cg15982099_ | -0.05 | 0.00 |
| PRTN3_cg19787694_ | -0.05 | 0.01 |
| PRTN3_cg19787694_ | -0.05 | 0.00 |
| AGTRAP_cg25467652_ | -0.05 | 0.00 |
| CXXC5_cg19628988_ | -0.05 | 0.00 |
| DENND3_cg14480530_ | -0.05 | 0.04 |
| DENND3_cg14480530_ | -0.05 | 0.01 |
| LTBR_cg19476647_ | -0.05 | 0.00 |
| SLC12A7_cg18997983_ | -0.05 | 0.00 |
| CXXC5_cg19628988_ | -0.05 | 0.00 |
| DENND3_cg14480530_ | -0.05 | 0.03 |
| DEF8_cg05185784_ | -0.04 | 0.00 |
| TUBGCP6_cg19851816_ | -0.04 | 0.00 |
| DOC2B_cg14984781_ | -0.04 | 0.04 |
| CXXC5_cg19628988_ | -0.04 | 0.00 |
| SPI1_cg15982099_ | -0.04 | 0.00 |
| NCLN_cg09462281_ | -0.04 | 0.03 |
| DENND3_cg14480530_ | -0.04 | 0.03 |
| DENND3_cg14480530_ | -0.04 | 0.03 |
| TSPOAP1_cg17525495_ | -0.04 | 0.00 |
| NCLN_cg09462281_ | -0.04 | 0.03 |
| CXXC5_cg19628988_ | -0.04 | 0.00 |
| MYL9_cg18660345_ | -0.04 | 0.01 |
| TSPOAP1_cg17525495_ | -0.04 | 0.00 |
| SPI1_cg15982099_ | -0.04 | 0.00 |
| PRTN3_cg19787694_ | -0.04 | 0.02 |
| TSPOAP1_cg17525495_ | -0.03 | 0.00 |
| LTBR_cg19476647_ | -0.03 | 0.00 |
| CXXC5_cg19628988_ | -0.03 | 0.00 |
| DENND3_cg14480530_ | -0.03 | 0.05 |
| CXXC5_cg19628988_ | -0.03 | 0.01 |
| CXXC5_cg19628988_ | -0.03 | 0.00 |
| TSPOAP1_cg17525495_ | -0.03 | 0.00 |
| TSPOAP1_cg17525495_ | -0.03 | 0.00 |
| SLC12A7_cg18997983_ | -0.03 | 0.01 |
| CXXC5_cg19628988_ | -0.03 | 0.00 |
| CXXC5_cg19628988_ | -0.03 | 0.00 |
| TSPOAP1_cg17525495_ | -0.03 | 0.01 |
| TSPOAP1_cg17525495_ | -0.03 | 0.00 |
| SLC12A7_cg18997983_ | -0.03 | 0.00 |
| TUBGCP6_cg19851816_ | -0.03 | 0.00 |
| EIF3A_cg08078028_ | -0.03 | 0.00 |
| LTBR_cg19476647_ | -0.03 | 0.00 |
| SPI1_cg15982099_ | -0.03 | 0.00 |
| MYL9_cg18660345_ | -0.03 | 0.01 |
| SLC12A7_cg18997983_ | -0.03 | 0.01 |
| CXXC5_cg19628988_ | -0.03 | 0.00 |
| LOC441666_cg27119456_ | -0.03 | 0.03 |
| NCLN_cg09462281_ | -0.03 | 0.03 |
| EIF3A_cg08078028_ | -0.02 | 0.02 |
| NCLN_cg09462281_ | -0.02 | 0.03 |
| MYL9_cg18660345_ | -0.02 | 0.02 |
| NCLN_cg09462281_ | -0.02 | 0.03 |
| EIF3A_cg08078028_ | -0.02 | 0.02 |
| LOC441666_cg27119456_ | -0.02 | 0.05 |
| SPI1_cg15982099_ | -0.02 | 0.00 |
| SLC12A7_cg18997983_ | -0.02 | 0.01 |
| LTBR_cg19476647_ | -0.02 | 0.00 |
| SPI1_cg15982099_ | -0.02 | 0.00 |
| LTBR_cg19476647_ | -0.02 | 0.00 |
| MYL9_cg18660345_ | -0.02 | 0.05 |
| SLC12A7_cg18997983_ | -0.01 | 0.01 |
| MYL9_cg18660345_ | -0.01 | 0.01 |
| CXXC5_cg19628988_ | -0.01 | 0.00 |
| LTBR_cg19476647_ | -0.01 | 0.00 |
| SPI1_cg15982099_ | -0.01 | 0.00 |
| SPI1_cg15982099_ | -0.01 | 0.03 |
| SPI1_cg15982099_ | -0.01 | 0.03 |
| SPI1_cg15982099_ | -0.01 | 0.00 |
| TUBGCP6_cg19851816_ | 0.00 | 0.04 |
| TUBGCP6_cg19851816_ | 0.00 | 0.05 |
| HSPG2_cg21272897_ | 0.00 | 0.01 |
| PIK3CD_cg23098018_ | 0.01 | 0.00 |
| PIK3CD_cg23098018_ | 0.01 | 0.00 |
| PIK3CD_cg23098018_ | 0.01 | 0.00 |
| PIK3CD_cg23098018_ | 0.01 | 0.00 |
| PIK3CD_cg23098018_ | 0.01 | 0.00 |
| PIK3CD_cg23098018_ | 0.01 | 0.00 |
| SERPINB9_cg01345354_ | 0.02 | 0.00 |
| PIK3CD_cg23098018_ | 0.02 | 0.00 |
| PIK3CD_cg23098018_ | 0.02 | 0.00 |
| PIK3CD_cg23098018_ | 0.02 | 0.00 |
| PIK3CD_cg23098018_ | 0.02 | 0.00 |
| PIK3CD_cg23098018_ | 0.02 | 0.00 |
| SERPINB9_cg01345354_ | 0.02 | 0.00 |
| PIK3CD_cg23098018_ | 0.02 | 0.00 |
| PIK3CD_cg23098018_ | 0.02 | 0.00 |
| SERPINB9_cg01345354_ | 0.02 | 0.00 |
| SERPINB9_cg01345354_ | 0.02 | 0.00 |
| SERPINB9_cg01345354_ | 0.02 | 0.00 |
| SERPINB9_cg01345354_ | 0.02 | 0.00 |
| PIK3CD_cg23098018_ | 0.02 | 0.00 |
| SERPINB9_cg01345354_ | 0.02 | 0.00 |
| SERPINB9_cg01345354_ | 0.02 | 0.00 |
| SERPINB9_cg01345354_ | 0.02 | 0.00 |
| SERPINB9_cg01345354_ | 0.02 | 0.00 |
| SERPINB9_cg01345354_ | 0.03 | 0.00 |
| RASA3_cg21261158_ | 0.03 | 0.02 |
| SERPINB9_cg01345354_ | 0.03 | 0.00 |
| RASA3_cg21261158_ | 0.03 | 0.03 |
| RASA3_cg21261158_ | 0.03 | 0.01 |
| RASA3_cg21261158_ | 0.03 | 0.02 |
| SERPINB9_cg01345354_ | 0.03 | 0.00 |
| DGKA_cg07679948_ | 0.03 | 0.00 |
| DGKA_cg07679948_ | 0.04 | 0.00 |
| DGKA_cg07679948_ | 0.04 | 0.00 |
| RASA3_cg21261158_ | 0.04 | 0.01 |
| RCAN3_cg25140783_ | 0.04 | 0.02 |
| DGKA_cg07679948_ | 0.04 | 0.00 |
| SERPINB9_cg01345354_ | 0.04 | 0.00 |
| RCAN3_cg25140783_ | 0.04 | 0.02 |
| DGKA_cg07679948_ | 0.04 | 0.00 |
| DGKA_cg07679948_ | 0.04 | 0.00 |
| DGKA_cg07679948_ | 0.04 | 0.00 |
| DGKA_cg07679948_ | 0.04 | 0.00 |
| RCAN3_cg25140783_ | 0.04 | 0.02 |
| RCAN3_cg25140783_ | 0.05 | 0.01 |
| RCAN3_cg25140783_ | 0.05 | 0.01 |
| RCAN3_cg25140783_ | 0.05 | 0.01 |
| DGKA_cg07679948_ | 0.05 | 0.00 |
| RCAN3_cg25140783_ | 0.05 | 0.01 |
| ZFP36L1_cg10099732_ | 0.05 | 0.00 |
| ZFP36L1_cg10099732_ | 0.05 | 0.00 |
| RCAN3_cg25140783_ | 0.05 | 0.01 |
| DGKA_cg07679948_ | 0.05 | 0.00 |
| ZFP36L1_cg10099732_ | 0.05 | 0.00 |
| ZFP36L1_cg10099732_ | 0.05 | 0.00 |
| ZFP36L1_cg10099732_ | 0.05 | 0.00 |
| ZFP36L1_cg10099732_ | 0.05 | 0.00 |
| ZFP36L1_cg10099732_ | 0.05 | 0.00 |
| RCAN3_cg25140783_ | 0.05 | 0.01 |
| ZFP36L1_cg10099732_ | 0.05 | 0.00 |
| ZFP36L1_cg10099732_ | 0.05 | 0.00 |
| ZFP36L1_cg08169020_ | 0.09 | 0.05 |
| ZFP36L1_cg08169020_ | 0.11 | 0.01 |
| NCLN_cg09462281_ | 0.11 | 0.01 |

MethylDiff: the difference between vaginal delivery and CDMR group in each locus

Table S4. The comparison of M-values between VD and CDMR group using multiple linear regression

| TargetID | p | fdr | p* | FDR* | p# | FDR# |
| --- | --- | --- | --- | --- | --- | --- |
| cg00051154 | 0.05 | 0.06 | 0.16 | 0.19 | 0.35 | 0.44 |
| cg00540941 | 0 | 0 | 0 | 0.02 | 0.01 | 0.07 |
| cg00616572 | 0.02 | 0.04 | 0.01 | 0.05 | 0.01 | 0.09 |
| cg01256139 | 0 | 0 | 0 | 0 | 0.01 | 0.07 |
| cg01412404 | 0.04 | 0.06 | 0.02 | 0.06 | 0.07 | 0.18 |
| cg01471923 | 0.16 | 0.17 | 0.35 | 0.37 | 0.75 | 0.78 |
| cg01823585 | 0.04 | 0.06 | 0.03 | 0.07 | 0.00 | 0.06 |
| cg02201753 | 0 | 0.02 | 0.02 | 0.05 | 0.06 | 0.17 |
| cg02444957 | 0.01 | 0.03 | 0.36 | 0.37 | 0.49 | 0.55 |
| cg02741327 | 0.05 | 0.06 | 0.04 | 0.07 | 0.19 | 0.30 |
| cg02902672 | 0.18 | 0.18 | 0.07 | 0.1 | 0.13 | 0.24 |
| cg02952913 | 0.01 | 0.03 | 0.04 | 0.07 | 0.10 | 0.21 |
| cg03024489 | 0.05 | 0.07 | 0.04 | 0.08 | 0.42 | 0.49 |
| cg03122926 | 0.01 | 0.03 | 0.04 | 0.07 | 0.03 | 0.14 |
| cg04246708 | 0 | 0.02 | 0 | 0 | 0.01 | 0.07 |
| cg04311686 | 0.05 | 0.07 | 0.21 | 0.23 | 0.36 | 0.45 |
| cg04368836 | 0.03 | 0.05 | 0.09 | 0.12 | 0.15 | 0.25 |
| cg04497611 | 0.04 | 0.06 | 0.01 | 0.05 | 0.05 | 0.17 |
| cg04628741 | 0.11 | 0.12 | 0.19 | 0.22 | 0.60 | 0.66 |
| cg04777551 | 0 | 0.01 | 0 | 0.01 | 0.01 | 0.07 |
| cg04824555 | 0 | 0.01 | 0.01 | 0.04 | 0.43 | 0.50 |
| cg05008296 | 0.05 | 0.06 | 0.26 | 0.28 | 0.39 | 0.48 |
| cg05023192 | 0.02 | 0.04 | 0.02 | 0.05 | 0.00 | 0.07 |
| cg05176970 | 0.05 | 0.06 | 0.03 | 0.07 | 0.18 | 0.28 |
| cg05308244 | 0.01 | 0.03 | 0.02 | 0.05 | 0.01 | 0.07 |
| cg05362314 | 0 | 0.01 | 0.02 | 0.06 | 0.26 | 0.36 |
| cg06063541 | 0.11 | 0.12 | 0.21 | 0.23 | 0.73 | 0.77 |
| cg06293782 | 0.01 | 0.03 | 0.07 | 0.1 | 0.01 | 0.07 |
| cg06334689 | 0.04 | 0.06 | 0.07 | 0.1 | 0.00 | 0.06 |
| cg06499415 | 0 | 0.01 | 0 | 0.02 | 0.04 | 0.14 |
| cg06546677 | 0 | 0.02 | 0.01 | 0.04 | 0.07 | 0.18 |
| cg06732825 | 0 | 0.02 | 0.04 | 0.07 | 0.80 | 0.82 |
| cg06924902 | 0.2 | 0.2 | 0.83 | 0.83 | 0.79 | 0.81 |
| cg07227024 | 0.25 | 0.25 | 0.11 | 0.13 | 0.07 | 0.18 |
| cg07240846 | 0.01 | 0.04 | 0.03 | 0.07 | 0.16 | 0.27 |
| cg07584620 | 0.06 | 0.07 | 0.18 | 0.21 | 0.75 | 0.78 |
| cg07743747 | 0 | 0.02 | 0.01 | 0.04 | 0.03 | 0.13 |
| cg07748255 | 0 | 0 | 0 | 0.01 | 0.33 | 0.43 |
| cg07837085 | 0 | 0 | 0 | 0 | 0.00 | 0.06 |
| cg07889790 | 0.14 | 0.15 | 0.2 | 0.22 | 0.66 | 0.71 |
| cg08102564 | 0.02 | 0.04 | 0 | 0.02 | 0.02 | 0.11 |
| cg08158105 | 0.04 | 0.06 | 0.02 | 0.05 | 0.97 | 0.97 |
| cg08506672 | 0.04 | 0.06 | 0.04 | 0.08 | 0.35 | 0.44 |
| cg08514194 | 0.02 | 0.04 | 0.04 | 0.07 | 0.17 | 0.28 |
| cg08617581 | 0.06 | 0.07 | 0.04 | 0.07 | 0.03 | 0.12 |
| cg08629394 | 0.01 | 0.04 | 0.02 | 0.05 | 0.00 | 0.06 |
| cg08786370 | 0 | 0.02 | 0 | 0.02 | 0.00 | 0.06 |
| cg08912652 | 0.01 | 0.04 | 0.03 | 0.06 | 0.11 | 0.22 |
| cg09157251 | 0.04 | 0.06 | 0.09 | 0.11 | 0.21 | 0.32 |
| cg09462281 | 0.03 | 0.05 | 0.03 | 0.06 | 0.20 | 0.30 |
| cg09854620 | 0.03 | 0.05 | 0.36 | 0.37 | 0.02 | 0.12 |
| cg09867002 | 0.77 | 0.78 | 0.29 | 0.31 | 0.20 | 0.31 |
| cg10277872 | 0.01 | 0.03 | 0.19 | 0.21 | 0.64 | 0.70 |
| cg10528424 | 0 | 0.01 | 0.03 | 0.06 | 0.06 | 0.17 |
| cg10680514 | 0.01 | 0.03 | 0.03 | 0.06 | 0.04 | 0.14 |
| cg10738003 | 0 | 0.01 | 0 | 0.02 | 0.00 | 0.06 |
| cg10911276 | 0.02 | 0.04 | 0.26 | 0.28 | 0.30 | 0.40 |
| cg10920758 | 0.03 | 0.05 | 0.05 | 0.08 | 0.28 | 0.38 |
| cg11019305 | 0.1 | 0.11 | 0.55 | 0.55 | 0.35 | 0.44 |
| cg11144986 | 0 | 0.01 | 0 | 0.02 | 0.09 | 0.20 |
| cg11251367 | 0 | 0.02 | 0.06 | 0.09 | 0.02 | 0.12 |
| cg11400162 | 0.02 | 0.04 | 0.22 | 0.24 | 0.54 | 0.60 |
| cg11495604 | 0 | 0.02 | 0.01 | 0.04 | 0.07 | 0.18 |
| cg11573390 | 0.06 | 0.07 | 0.05 | 0.09 | 0.07 | 0.18 |
| cg11585022 | 0.02 | 0.04 | 0.08 | 0.1 | 0.02 | 0.11 |
| cg11680857 | 0.06 | 0.08 | 0.01 | 0.04 | 0.05 | 0.17 |
| cg11723923 | 0.06 | 0.07 | 0.04 | 0.07 | 0.40 | 0.48 |
| cg11768182 | 0 | 0.01 | 0 | 0.02 | 0.02 | 0.12 |
| cg11784298 | 0 | 0.02 | 0 | 0.02 | 0.00 | 0.06 |
| cg11844537 | 0.16 | 0.17 | 0.29 | 0.31 | 0.22 | 0.32 |
| cg11872966 | 0.05 | 0.06 | 0.11 | 0.14 | 0.10 | 0.21 |
| cg11882358 | 0.04 | 0.06 | 0.05 | 0.08 | 0.23 | 0.32 |
| cg11897887 | 0.04 | 0.06 | 0.06 | 0.09 | 0.43 | 0.50 |
| cg11987751 | 0.01 | 0.03 | 0 | 0.02 | 0.05 | 0.17 |
| cg12031275 | 0.04 | 0.06 | 0.02 | 0.06 | 0.22 | 0.32 |
| cg12303247 | 0 | 0.02 | 0.01 | 0.04 | 0.06 | 0.17 |
| cg12474444 | 0 | 0.01 | 0 | 0.02 | 0.01 | 0.07 |
| cg12475092 | 0.01 | 0.03 | 0.03 | 0.06 | 0.15 | 0.25 |
| cg13064658 | 0 | 0.01 | 0 | 0.03 | 0.00 | 0.06 |
| cg13066461 | 0.04 | 0.06 | 0.26 | 0.28 | 0.07 | 0.18 |
| cg13111532 | 0.01 | 0.03 | 0.01 | 0.04 | 0.11 | 0.22 |
| cg13183651 | 0.03 | 0.05 | 0.06 | 0.09 | 0.35 | 0.44 |
| cg13295089 | 0.02 | 0.04 | 0.01 | 0.04 | 0.17 | 0.28 |
| cg13488137 | 0.05 | 0.06 | 0.07 | 0.1 | 0.17 | 0.28 |
| cg13573375 | 0.05 | 0.07 | 0.12 | 0.15 | 0.23 | 0.33 |
| cg13595143 | 0 | 0.02 | 0.01 | 0.04 | 0.15 | 0.26 |
| cg13641645 | 0.05 | 0.06 | 0.22 | 0.24 | 0.15 | 0.26 |
| cg13653328 | 0.03 | 0.05 | 0.06 | 0.09 | 0.32 | 0.42 |
| cg13740636 | 0.04 | 0.06 | 0.01 | 0.03 | 0.10 | 0.21 |
| cg13854498 | 0.03 | 0.05 | 0 | 0.02 | 0.01 | 0.07 |
| cg14195178 | 0.02 | 0.04 | 0.01 | 0.04 | 0.04 | 0.15 |
| cg14279361 | 0.02 | 0.04 | 0.05 | 0.08 | 0.10 | 0.21 |
| cg14341177 | 0.01 | 0.03 | 0.01 | 0.04 | 0.04 | 0.15 |
| cg14494781 | 0.01 | 0.03 | 0 | 0.02 | 0.01 | 0.07 |
| cg14799809 | 0.19 | 0.2 | 0.09 | 0.12 | 0.09 | 0.20 |
| cg15029183 | 0.04 | 0.06 | 0.02 | 0.05 | 0.03 | 0.12 |
| cg15135990 | 0 | 0.02 | 0.01 | 0.04 | 0.06 | 0.17 |
| cg15161973 | 0.17 | 0.18 | 0.78 | 0.79 | 0.66 | 0.71 |
| cg15226348 | 0.05 | 0.06 | 0.11 | 0.13 | 0.91 | 0.92 |
| cg15293181 | 0 | 0.02 | 0.09 | 0.12 | 0.08 | 0.19 |
| cg15460035 | 0.03 | 0.05 | 0.17 | 0.2 | 0.73 | 0.77 |
| cg15567368 | 0.08 | 0.09 | 0.1 | 0.12 | 0.18 | 0.29 |
| cg15837943 | 0.01 | 0.03 | 0.05 | 0.08 | 0.04 | 0.15 |
| cg15971518 | 0.02 | 0.04 | 0.03 | 0.07 | 0.14 | 0.25 |
| cg16248432 | 0.15 | 0.16 | 0.11 | 0.14 | 0.12 | 0.23 |
| cg16261114 | 0 | 0 | 0 | 0 | 0.01 | 0.07 |
| cg16471877 | 0 | 0.02 | 0.01 | 0.04 | 0.10 | 0.21 |
| cg16886051 | 0.12 | 0.13 | 0.2 | 0.22 | 0.38 | 0.47 |
| cg17009574 | 0.01 | 0.03 | 0.01 | 0.04 | 0.02 | 0.10 |
| cg17373649 | 0.01 | 0.03 | 0.03 | 0.06 | 0.40 | 0.48 |
| cg17628491 | 0.06 | 0.08 | 0.04 | 0.07 | 0.07 | 0.18 |
| cg17759595 | 0.02 | 0.04 | 0.06 | 0.09 | 0.12 | 0.22 |
| cg17813879 | 0.01 | 0.03 | 0.09 | 0.12 | 0.22 | 0.32 |
| cg17839758 | 0.04 | 0.06 | 0.07 | 0.1 | 0.20 | 0.31 |
| cg18486102 | 0 | 0.01 | 0 | 0.02 | 0.01 | 0.07 |
| cg18500967 | 0.03 | 0.05 | 0.04 | 0.07 | 0.10 | 0.21 |
| cg18949721 | 0.05 | 0.06 | 0.15 | 0.18 | 0.09 | 0.21 |
| cg19236675 | 0.01 | 0.03 | 0.01 | 0.05 | 0.08 | 0.19 |
| cg19556341 | 0 | 0 | 0 | 0.02 | 0.02 | 0.10 |
| cg19591135 | 0.02 | 0.04 | 0.05 | 0.08 | 0.40 | 0.48 |
| cg19653246 | 0.03 | 0.05 | 0.05 | 0.08 | 0.09 | 0.20 |
| cg19816075 | 0.02 | 0.04 | 0.01 | 0.04 | 0.03 | 0.14 |
| cg20089799 | 0.01 | 0.04 | 0 | 0.01 | 0.01 | 0.07 |
| cg20370184 | 0.01 | 0.03 | 0.16 | 0.19 | 0.26 | 0.36 |
| cg20848488 | 0.01 | 0.03 | 0.04 | 0.08 | 0.07 | 0.18 |
| cg21211688 | 0 | 0.02 | 0.02 | 0.06 | 0.14 | 0.25 |
| cg21272897 | 0.01 | 0.04 | 0.01 | 0.03 | 0.04 | 0.14 |
| cg21388339 | 0.01 | 0.03 | 0.05 | 0.09 | 0.06 | 0.17 |
| cg21442271 | 0.02 | 0.04 | 0.06 | 0.09 | 0.11 | 0.22 |
| cg21700663 | 0.01 | 0.03 | 0.01 | 0.04 | 0.13 | 0.24 |
| cg21932934 | 0.01 | 0.03 | 0.03 | 0.06 | 0.33 | 0.43 |
| cg21974656 | 0.01 | 0.04 | 0.01 | 0.04 | 0.04 | 0.14 |
| cg22112152 | 0.07 | 0.09 | 0.01 | 0.05 | 0.06 | 0.17 |
| cg22238209 | 0.02 | 0.04 | 0.02 | 0.06 | 0.17 | 0.28 |
| cg22373097 | 0.24 | 0.24 | 0.24 | 0.26 | 0.52 | 0.58 |
| cg22543924 | 0.08 | 0.09 | 0.14 | 0.17 | 0.35 | 0.44 |
| cg22681945 | 0.08 | 0.09 | 0.07 | 0.1 | 0.88 | 0.90 |
| cg22741595 | 0.11 | 0.12 | 0.14 | 0.17 | 0.12 | 0.22 |
| cg22804805 | 0.02 | 0.04 | 0.01 | 0.04 | 0.09 | 0.21 |
| cg22836174 | 0 | 0 | 0 | 0.02 | 0.05 | 0.17 |
| cg22931151 | 0.03 | 0.05 | 0.09 | 0.12 | 0.18 | 0.29 |
| cg22933800 | 0.03 | 0.05 | 0.01 | 0.04 | 0.03 | 0.12 |
| cg22971153 | 0.02 | 0.04 | 0.03 | 0.07 | 0.14 | 0.25 |
| cg23098789 | 0.02 | 0.04 | 0 | 0.02 | 0.00 | 0.06 |
| cg23130832 | 0.1 | 0.11 | 0.08 | 0.11 | 0.28 | 0.38 |
| cg23159165 | 0.01 | 0.03 | 0.01 | 0.04 | 0.04 | 0.14 |
| cg23333490 | 0.02 | 0.04 | 0.01 | 0.04 | 0.04 | 0.15 |
| cg23681001 | 0.06 | 0.07 | 0.06 | 0.09 | 0.44 | 0.50 |
| cg23825480 | 0 | 0 | 0 | 0.01 | 0.45 | 0.52 |
| cg24137123 | 0.08 | 0.09 | 0.07 | 0.1 | 0.06 | 0.18 |
| cg24441810 | 0 | 0 | 0 | 0 | 0.26 | 0.36 |
| cg24629711 | 0.08 | 0.09 | 0.42 | 0.42 | 0.93 | 0.94 |
| cg24739935 | 0.02 | 0.04 | 0.05 | 0.09 | 0.01 | 0.07 |
| cg24844518 | 0.02 | 0.04 | 0.03 | 0.07 | 0.02 | 0.12 |
| cg25049941 | 0.08 | 0.09 | 0.4 | 0.41 | 0.35 | 0.44 |
| cg25649515 | 0.04 | 0.06 | 0.06 | 0.1 | 0.18 | 0.28 |
| cg25674027 | 0.02 | 0.04 | 0.06 | 0.09 | 0.58 | 0.64 |
| cg25830305 | 0.04 | 0.06 | 0.23 | 0.25 | 0.41 | 0.49 |
| cg26337497 | 0.07 | 0.08 | 0.03 | 0.06 | 0.07 | 0.18 |
| cg26465155 | 0.03 | 0.05 | 0.07 | 0.1 | 0.03 | 0.14 |
| cg27244972 | 0.04 | 0.06 | 0.06 | 0.09 | 0.67 | 0.72 |
| cg27346510 | 0.02 | 0.04 | 0.06 | 0.09 | 0.12 | 0.22 |
| cg27433479 | 0.01 | 0.03 | 0.04 | 0.08 | 0.43 | 0.50 |
| cg27468880 | 0 | 0.02 | 0.02 | 0.06 | 0.25 | 0.35 |
| cg27577781 | 0.02 | 0.04 | 0.1 | 0.13 | 0.63 | 0.69 |

* Multiple linear regression models were used to avoid confounding effects, adjusting the potential confounders in Table 1.

# Multiple linear regression models were used to avoid confounding effects, adjusting the potential confounders in Table 1 and the proportions of cell types.

Table S5. The estimate proportions of cell type in cord blood

| Sample | CD8T | CD4T | NK | Bcell | Mono | Gran | nRBC |
| --- | --- | --- | --- | --- | --- | --- | --- |
| 1 | 0.00 | 0.15 | 0.00 | 0.06 | 0.09 | 0.47 | 0.23 |
| 2 | 0.02 | 0.12 | 0.06 | 0.05 | 0.11 | 0.65 | 0.00 |
| 3 | 0.01 | 0.07 | 0.00 | 0.00 | 0.03 | 0.80 | 0.08 |
| 4 | 0.03 | 0.13 | 0.01 | 0.01 | 0.11 | 0.69 | 0.03 |
| 5 | 0.05 | 0.19 | 0.05 | 0.05 | 0.13 | 0.48 | 0.07 |
| 6 | 0.01 | 0.09 | 0.02 | 0.03 | 0.10 | 0.72 | 0.04 |
| 7 | 0.00 | 0.07 | 0.04 | 0.02 | 0.12 | 0.73 | 0.03 |
| 8 | 0.00 | 0.05 | 0.03 | 0.01 | 0.04 | 0.82 | 0.08 |
| 9 | 0.00 | 0.12 | 0.01 | 0.02 | 0.08 | 0.76 | 0.01 |
| 10 | 0.02 | 0.15 | 0.00 | 0.04 | 0.03 | 0.74 | 0.00 |
| 11 | 0.02 | 0.13 | 0.04 | 0.01 | 0.05 | 0.74 | 0.00 |
| 12 | 0.02 | 0.15 | 0.00 | 0.00 | 0.07 | 0.64 | 0.10 |
| 13 | 0.04 | 0.17 | 0.02 | 0.07 | 0.06 | 0.62 | 0.01 |
| 14 | 0.00 | 0.14 | 0.05 | 0.03 | 0.09 | 0.67 | 0.00 |
| 15 | 0.00 | 0.10 | 0.05 | 0.04 | 0.06 | 0.71 | 0.03 |
| 16 | 0.00 | 0.10 | 0.09 | 0.02 | 0.17 | 0.58 | 0.00 |
| 17 | 0.00 | 0.10 | 0.03 | 0.01 | 0.05 | 0.76 | 0.05 |
| 18 | 0.03 | 0.13 | 0.02 | 0.01 | 0.09 | 0.69 | 0.00 |
| 19 | 0.00 | 0.07 | 0.01 | 0.01 | 0.03 | 0.82 | 0.04 |
| 20 | 0.05 | 0.14 | 0.01 | 0.11 | 0.06 | 0.63 | 0.02 |
| 21 | 0.00 | 0.07 | 0.03 | 0.02 | 0.08 | 0.80 | 0.01 |
| 22 | 0.00 | 0.12 | 0.01 | 0.02 | 0.06 | 0.75 | 0.03 |
| 23 | 0.01 | 0.10 | 0.01 | 0.04 | 0.07 | 0.77 | 0.01 |
| 24 | 0.00 | 0.06 | 0.03 | 0.00 | 0.04 | 0.88 | 0.00 |
| 25 | 0.00 | 0.09 | 0.02 | 0.04 | 0.08 | 0.77 | 0.03 |
| 26 | 0.02 | 0.22 | 0.02 | 0.02 | 0.11 | 0.61 | 0.02 |
| 27 | 0.03 | 0.22 | 0.07 | 0.04 | 0.07 | 0.56 | 0.02 |
| 28 | 0.01 | 0.13 | 0.06 | 0.06 | 0.08 | 0.64 | 0.02 |
| 29 | 0.00 | 0.06 | 0.03 | 0.03 | 0.07 | 0.58 | 0.24 |
| 30 | 0.01 | 0.08 | 0.04 | 0.01 | 0.03 | 0.78 | 0.07 |
| 31 | 0.03 | 0.13 | 0.01 | 0.03 | 0.06 | 0.70 | 0.05 |
| 32 | 0.01 | 0.12 | 0.07 | 0.03 | 0.12 | 0.65 | 0.00 |
| 33 | 0.01 | 0.15 | 0.01 | 0.02 | 0.07 | 0.73 | 0.00 |
| 34 | 0.05 | 0.15 | 0.01 | 0.05 | 0.06 | 0.68 | 0.00 |
| 35 | 0.07 | 0.22 | 0.02 | 0.03 | 0.05 | 0.59 | 0.03 |
| 36 | 0.06 | 0.30 | 0.02 | 0.04 | 0.06 | 0.48 | 0.02 |
| 37 | 0.02 | 0.13 | 0.01 | 0.04 | 0.09 | 0.67 | 0.05 |
| 38 | 0.05 | 0.16 | 0.07 | 0.03 | 0.08 | 0.59 | 0.05 |
| 39 | 0.02 | 0.12 | 0.03 | 0.05 | 0.12 | 0.64 | 0.03 |
| 40 | 0.00 | 0.20 | 0.01 | 0.05 | 0.06 | 0.67 | 0.01 |
| 41 | 0.02 | 0.09 | 0.00 | 0.04 | 0.11 | 0.74 | 0.02 |
| 42 | 0.04 | 0.24 | 0.02 | 0.08 | 0.07 | 0.57 | 0.00 |
| 43 | 0.00 | 0.09 | 0.03 | 0.04 | 0.06 | 0.76 | 0.02 |
| 44 | 0.04 | 0.16 | 0.04 | 0.06 | 0.10 | 0.59 | 0.00 |
| 45 | 0.04 | 0.20 | 0.02 | 0.11 | 0.12 | 0.49 | 0.03 |
| 46 | 0.05 | 0.23 | 0.01 | 0.05 | 0.05 | 0.54 | 0.06 |
| 47 | 0.05 | 0.25 | 0.01 | 0.03 | 0.02 | 0.61 | 0.03 |
| 48 | 0.01 | 0.27 | 0.02 | 0.06 | 0.02 | 0.62 | 0.00 |
| 49 | 0.02 | 0.15 | 0.02 | 0.04 | 0.05 | 0.72 | 0.04 |
| 50 | 0.05 | 0.13 | 0.04 | 0.06 | 0.09 | 0.67 | 0.00 |
| 51 | 0.01 | 0.16 | 0.02 | 0.06 | 0.05 | 0.71 | 0.01 |
| 52 | 0.05 | 0.20 | 0.00 | 0.03 | 0.07 | 0.58 | 0.09 |
| 53 | 0.00 | 0.13 | 0.02 | 0.06 | 0.08 | 0.71 | 0.00 |
| 54 | 0.02 | 0.18 | 0.02 | 0.02 | 0.05 | 0.68 | 0.04 |
| 55 | 0.00 | 0.19 | 0.01 | 0.07 | 0.06 | 0.64 | 0.04 |
| 56 | 0.04 | 0.17 | 0.03 | 0.06 | 0.09 | 0.63 | 0.01 |
| 57 | 0.03 | 0.28 | 0.07 | 0.09 | 0.02 | 0.52 | 0.00 |
| 58 | 0.00 | 0.22 | 0.01 | 0.06 | 0.08 | 0.63 | 0.00 |
| 59 | 0.00 | 0.18 | 0.02 | 0.06 | 0.05 | 0.66 | 0.04 |
| 60 | 0.04 | 0.25 | 0.04 | 0.05 | 0.08 | 0.52 | 0.03 |

Table S6. Differentially methylated CpG sites between VD and CDMR group from the Methylation Chip analyzed by M values, under the threshold difference in the mean β value |Δβ| ≥ 10%

| TargetID | MethylDiff | CHR | UCSC_REFGENE_NAME | FDR |
| --- | --- | --- | --- | --- |
| cg23079808 | -0.10 | 12 | LTBR | 0.00 |
| cg02058408 | 0.12 | 17 |  | 0.00 |
| cg11144986 | -0.11 | 12 |  | 0.01 |
| cg06499415 | -0.13 | 9 |  | 0.01 |

MethylDiff: the difference between vaginal delivery and CDMR group in each locus

CHR: Chromosome

UCSC_REFGENE_NAME: UCSC Reference Gene Name

**Caption of Figures**

Figure S1. A volcano plot of lg-transformed P values vs differences in DNA methylation (b-value) between VD and CDMR group. Horizontal and vertical lines denote thresholds for the definition of differentially methylated positions (>10% difference in methylation, P < 0.05). Red dots indicate the hypermethylated differentially methylated positions (DMPs) comparing VD with CDMR, blue dots indicate the hypomethylated DMPs.
