# Supplementary figures and images for: The impact of cesarean delivery on infant DNA methylation
Source: BMC Pregnancy Childbirth. 2021 Mar 30;21:265. doi: 10.1186/s12884-021-03748-y (PMC8011183; doi:10.1186/s12884-021-03748-y)

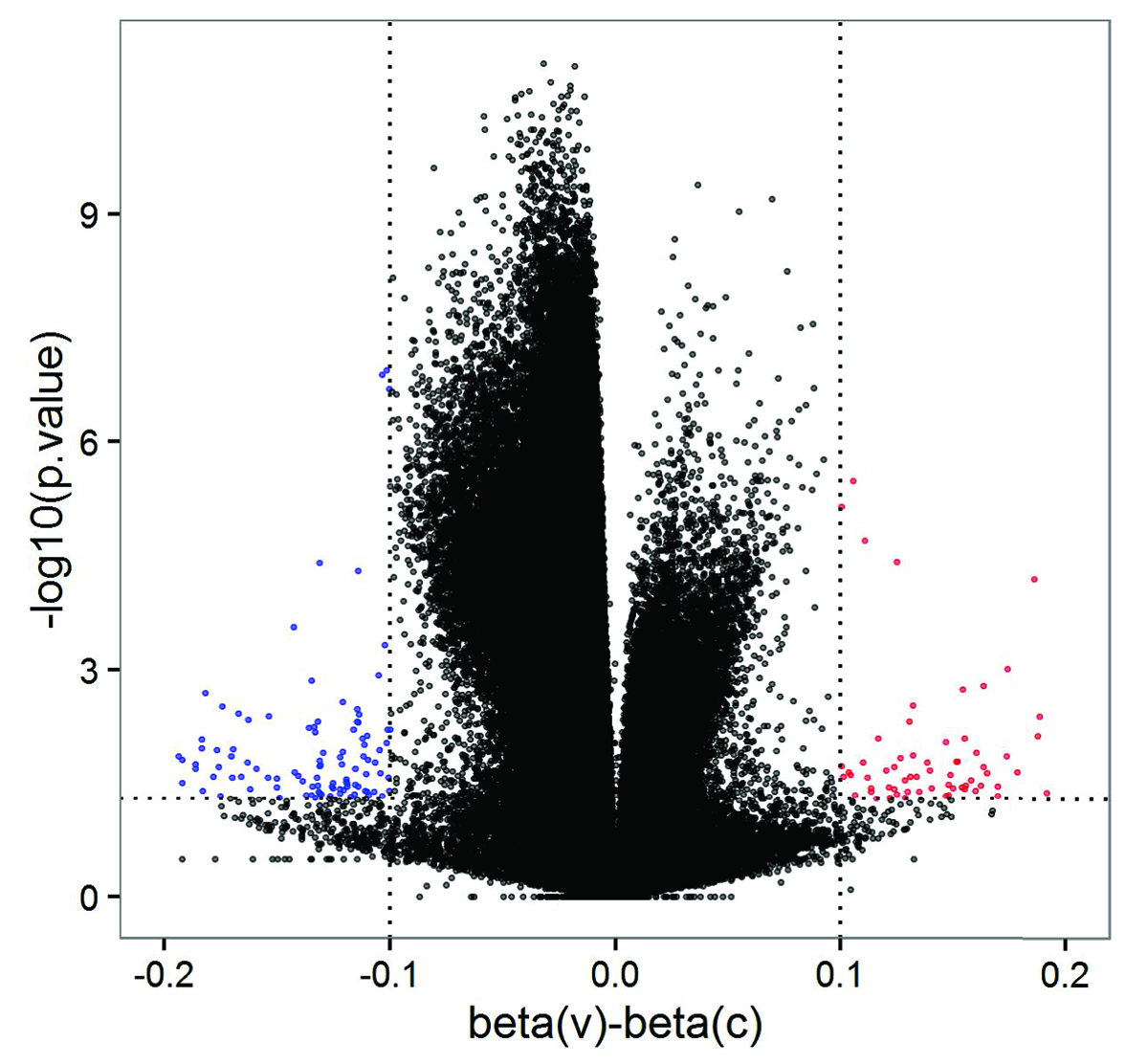

Supplement: Supplementary file 1 — Additional file 1 Table S1. Information of primers used in targeted bisulfite sequencing assay. Table S2. Differentially methylated loci between vaginal delivery and CDMR group from the Methylation Chip. Table S3. Differentially methylated loci between vaginal delivery and CDMR group from targeted bisulfite sequencing assay. Table S4. The comparison of M-values between VD and CDMR group using multiple linear regression. Table S5. The estimate proportions of cell type in cord blood. Table S6. Differentially methylated CpG sites between VD and CDMR group from the Methylation Chip analyzed by M values, under the threshold difference in the mean β value |Δβ| ≥ 10%. Fig. S1. A volcano plot of lg-transformed P values vs differences in DNA methylation (b-value) between VD and CDMR group. [file 12884_2021_3748_MOESM1_ESM.zip › Supplementary figure-The impact of cesarean delivery on infant DNA methylation.tif]
